# Circulating microRNAs in Fabry Disease

**DOI:** 10.1038/s41598-019-51805-6

**Published:** 2019-10-24

**Authors:** Ke Xiao, Dongchao Lu, Jeannine Hoepfner, Laura Santer, Shashi Gupta, Angelika Pfanne, Sabrina Thum, Malte Lenders, Eva Brand, Peter Nordbeck, Thomas Thum

**Affiliations:** 1 Institute of Molecular and Translational Therapeutic Strategies (IMTTS), Hannover Medical School, Hannover, Germany; 2 Internal Medicine D, Department of Nephrology, Hypertension and Rheumatology, University Hospital Muenster, Muenster, Germany; 3 Julius Maximilians-Universität, Department of Cardiology, Würzburg, Germany; 4 Excellence Cluster REBIRTH, Hannover Medical School, Hannover, Germany

**Keywords:** Diagnostic markers, Cardiovascular biology

## Abstract

Fabry disease is an X-linked deficiency of the lysosomal hydrolase alpha-galactosidase A (alpha-Gal). This results in an accumulation of globotriaosylceramide (GL-3/Gb3) in a variety of cells with subsequent functional impairment. The continuous progress of FD often leads to decreased quality of life and premature death caused by multi-organic complications. The overall aim of our study was to determine the amount of circulating miRNAs in Fabry patients and to test whether ERT would alter the level of individual circulating miRNAs. We used miRNA sequencing by the HTG EdgeSeq System to identify the circulating miRNA pool from Fabry patients with and without enzyme replacement therapy (n = 6). In total, 296 miRNAs in serum of patients were identified. Among them 9 miRNAs were further evaluated in extra serum samples (n = 31) using real-time qPCR and 6 of them showed significant differential expression. The resulting miRNA pattern may help to better understand mechanisms involved in the beneficial effects of ERT and these new miRNA markers could help to estimate the efficacy of ERT or to identify Fabry patients with specific need for ERT.

## Introduction

Fabry disease (FD) is an X-chromosome linked disorder caused by mutations in gene GLA coding for alpha-galactosidase-A enzyme (alpha-Gal). The enzyme activity deficiency that results in an accumulation of globotriaosylceramide (GL-3/Gb3) in a variety of cells often leads to subsequent functional impairment^1^. The initial manifestations of Fabry disease usually start in adolescence stage of life, including neuropathic pain (acroparesthesia) and abdominal discomfort^2^. The continuous progress of FD results in decreased quality of life and premature death caused by multi-organic complications^3,4^. As a specific treatment, Enzyme replacement therapy (ERT) has been shown to stabilize and reduce many signs and symptoms of Fabry disease^5–7^. More recently, oral chaperone therapy was shown to be also effective in selected Fabry patients depending on the underlying gene mutation^8^. Of clinical importance is the fact that early diagnosis and treatment in the disease course may delay or prevent the progression towards irreversible organ dysfunction and the consequent life-threatening complications. This is sometimes difficult due to the high variability of the severity and multi-organ system involvement in Fabry disease^9^. Next to the clinical features, enzyme activity tests and DNA sequencing are available to confirm the diagnosis^10^. Globotriaosylsphingosine (LysoGb3) serves as a useful biomarker to improve the diagnosis of heterozygous Fabry disease for therapeutic evaluation and monitoring^11^. In addition, circulating serum proteins in the blood of Fabry patients may help to get more information about the underlying pathophysiological mechanisms^12^.

Recently, a group of small RNA molecules known as microRNAs (miRNAs) have been proved to play essential roles in the cardiac function^13,14^. Moreover, the expression levels of miRNAs that present in circulating fluid usually differ between healthy and diseased patients. Although the underlying biological function and origin of these circulating molecules remains unclear, miRNAs are becoming potential biomarkers for early stage diagnosis and treatment response^15^. The overall aim of this study was to determine the amount of circulating miRNAs in Fabry patients and to test whether ERT would alter the level of individual circulating miRNAs.

## Materials and Methods

**Figure 1:**
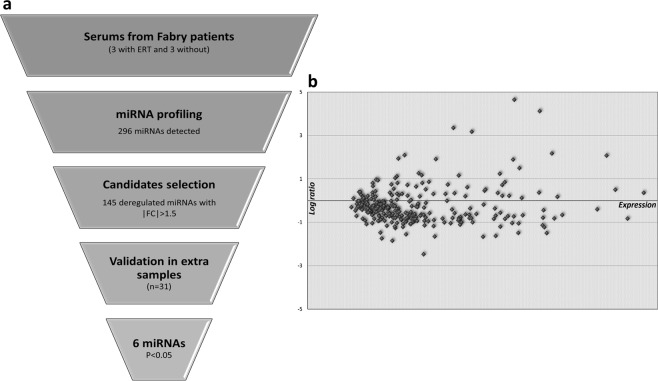
Screening strategy and the global expression pattern of miRNAs in the serum of Fabry patients. (**a**) Schematic strategy for identification and validation of the deregulated miRNAs. (**b**) The MA-plot illustrates the log transformed fold change (y-axis) of miRNA expression between patients with and without ERT versus normalized expression level (x-axis) of the 296 miRNAs detected by global screening.

We used RNA sequencing technologies to identify a specific miRNA pattern in serum of Fabry patients (Fig. 1). The inclusion criteria for this study were based on a confirmed mutation within the GLA gene and a classical or non-classical/late-onset clinical phenotype. The diagnostic criteria for FD were based on the recent publication by Biegstraaten *et al*.^16^: a genetically confirmed GLA mutation leading to deficient AGAL activity combined by one or more characteristic FD signs/symptoms, or an increase of plasma lyso-Gb3, or an additional family member with a definite FD diagnosis. Clinical characteristics of recruited patients were summarized in Table 1.

**Table 1:** Overview of patient groups.

Case Nr.	ERT	Age at visit	Gender	Mutation type	Mutation	MSSI^a^ score	Classical/non-classical	AGAL activity^b^	lyso-Gb3^c^ (ng/ml)	IVSd^d^ (mm)	NYHA class	eGFR^e^	FD-related pain
**Screening by RNA-seq**													
S1	with	50	M	missense	p.R112C	54	classical	5	NA	14	III	8	+
S2	with	47	M	missense	p.L129P	45	classical	2.5	21.2	15	I	59	+
S3	with	45	M	frameshift	fs 66X	62	classical	NA	NA	16	III	Haemodialysis	+
S4	without	42	F	frameshift	fs 268X	3	classical	55	7.52	10	I	98	
S5	without	19	M	frameshift	fs 268X	22	classical	10	121	14	I	132	+
S6	without	47	F	missense	p.W236C	16	classical	57.5	6.73	14	I	95	
**Validation by qPCR**													
P1	with	17	M	splice site	IVS2+1 G > A	4	classical	3	19.9	8		136	+
P2	with	22	M	splice site	IVS2+1 G > T	13	classical	3	31.6	7	I	126	+
P3	with	28	M	splice site	IVS5 +3 A > T	22	classical	24	28.4	9	I	49	+
P4	with	34	M	missense	p.L45P	8	classical	15	37.6	13		114	+
P5	with	39	M	missense	p.C94S	6	classical	9	21.5	15	I	114	+
P6	with	39	M	nonsense	p.W399X	37	classical	5	107	11	II	38	
P7	with	40	M	missense	p.G325S	19	classical	23	6.7	18	I	50	
P8	with	47	M	missense	p.D170N	21	classical	12	32.1	17	II	106	+
P9	with	49	M	missense	p.P259R	33	classical	22	18.6	15	I	71	+
P10	with	50	M	missense	p.K213M	19	classical	32	10.4	13	I	32	+
P11	with	54	M	splice site	IVS3 +1 G > A	51	classical	5	22.8	18	III	29	+
P12	with	57	M	missense	p.N215S	14	non-classical	12.5	5.4	10	II	83	
P13	with	57	F	frameshift	fs 338×	14	classical	77.5	8	9	II	90	
P14	with	62	M	missense	p.C172G	34	classical	<1	48.7	17	IV	26	+
P15	with	64	M	missense	p.N215S	15	late-onset	4	3.7	14	II	96	
P16	with	73	F	missense	p.D136E	33	classical	37.5	11.9	11	II	56	
P17	with	76	F	missense	p.G325S	29	non-classical	57.5	9	15	III	31	
P18	without	18	M	missense	p.M267T	10	classical	35	NA	13		130	
P19	without	23	M	nonsense	p.Y151X	4	classical	12	197	10		125	+
P20	without	32	M	missense	p.L45P	23	classical	<1	48.8	13		123	+
P21	without	34	M	missense	p.G35E	11	classical	4	45.3	10	I	112	
P22	without	35	M	nonsense	p.W349X	21	classical	12	164	13	I	105	+
P23	without	43	M	missense	p.W162G	21	classical	6	33.9	20	III	77	+
P24	without	45	F	missense	p.D136E	13	classical	60	5.6	8	I	89	
P25	without	46	M	nonsense	p.Y216X	25	classical	8	173	15	I	82	+
P26	without	46	F	missense	p.W287S	28	classical	57.5	17.4	15	I	120	
P27	without	49	M	missense	p.W162C	22	classical	12	25.4	36	III	79	
P28	without	53	M	missense	p.R342Q	30	classical	12	120	14	II	38	+
P29	without	56	M	missense	p.I242V	21	non-classical	87	0.6	20	I	117	+
P30	without	57	M	missense	p.L68F	41	classical	5	150	18	III	100	+
P31	without	64	M	missense	p.R301Q	17	classical	28	26.7	12	II	66	

^a^The Mainz Severity Score Index.

^b^The AGAL activities were determined from leukocytes (normal value >32 nmol MU/h/mg protein) or dried blood spots (normal value > 2.5 µ mol/l/h), patients’ AGAL activities are expressed as % of individual AGAL normal values.

^c^The normal level of lyso-Gb3 in this study is between 0.9–1.9 ng/ml or lower.

^d^Interventricular septal thickness at end-diastole (mm).

^e^Estimated glomerular filtration rate calculated using serum creatinine and the CKD-EPI equation.

In brief, the HTG EdgeSeq system was first utilized to identify and quantify the expression of regulated miRNAs directly in serum of 6 Fabry patients with and without ERT. After the bioinformatic analysis of reads data generated from the high-throughput platform, selected miRNA candidates were further evaluated in extra 31 serum samples (Table 1) from 17 patients with ERT and 14 without. Recruited patients for this study or their parents/legal guardian have signed informed consent before participation. The study has been approved by the local ethical committees of the University Hospital of Münster and the University Hospital of Würzburg therefore were performed in accordance with the Helsinki declaration.

The HTG EdgeSeq system utilizes a novel target capture and library prep chemistry that enables easy and fast use of next-generation sequencers such as Illumina for transcriptome analysis including miRNAs. The automated extraction-free chemistry of HTG EdgeSeq reduces the input requirement of samples and eliminates biases due to RNA extraction and library preparation. This increases the reproducibility of libraries prepared from raw precious samples such as serum used in this study. The raw read counts data was then generated by combined NGS sequencer for bioinformatic analyses and the selected candidates were validated with a miRNA-specific RT-qPCR method in extra samples as described previously^17^. All experiments were performed according to corresponding manufacturer’s protocols or instructions.

### MicroRNA Sequencing and quantification

15 µl serums from each of 6 patients including 3 treated with ERT for more than one year and 3 without ERT were incubated with HTG lysis buffer and Proteinase K (Ambion) at 20 °C for 2 hours. The sample plates were then loaded into an HTG Edgeseq Processor. After the automated preparation process, library were prepared with TruSeq Small RNA Prep kit (Illumina) according to the manufacturer’s instruction. Single-end reads of 51 bp in length were then sequenced on an Illumina GAIIx instrument. For expression level quantification, trimmed reads were mapped to the genome reference (hg19) allowing one mismatch and quantified applying Avadis NGS software (v1.4). Reads mapped to multiple locations in the genome were removed from further quantification. Annotation from miRBase v20 were used to designate reference mapped reads to miRNAs.

### Data normalization and differential expression analysis

A scaling factor for each sample ***і***, is obtained for each gene ***g*** and samples ***m***. The scaling factor ***S***_***i***_, is the median gene level expression value for each sample-gene count adjusted by the geometric mean over all genes. Note that any genes without expression over all samples are necessarily excluded from this scaling calculation. The formula for the scaling factor for the ***i***^***th***^ sample can be written as Eq. (1):

1 $${S}_{i}=media{n}_{g}\frac{{r}_{gi}}{\,({\prod }_{v=1}^{m}{r}_{gv}){}^{1/m}\,}$$

Where, ***r***_***gi***_ is the raw count for the ***i***^***th***^ sample and ***g***^***th***^ gene.

The scaling factor is then used to modify the original read counts to obtain the normalized count value ${r}_{gi}^{nor}$ in Eq. (2):

2 $${r}_{gi}^{nor}=\frac{{r}_{gi}}{{S}_{i}}$$

The normalized data, ${{\boldsymbol{r}}}_{gi}^{nor}$, can then be used for differential expression analysis. This method is included as part of the DESeq2 package when using Bioconductor and the R statistical package. Information about this method and the used packages has been described earlier^18,19^. After normalization, unpaired t-test was performed to detect the deregulated miRNAs. To exclude the very low/unstable expressed miRNAs in each condition, with or without ERT treatment, any miRNA shows no expression in at least 2 samples out of 6 were removed from further analysis.

### Candidate microRNAs validation via Real-Time PCR

From the RNA-seq based profiling results we selected 9 miRNAs for validation in serum samples collected from extra 31 Fabry patients (Table 1). Specifically, the serum samples were centrifuged at 2000g for 10 min at room temperature, from which the liquid supernatant were obtained and stored at −80 °C. MiRNA were then isolated using the miRNeasy Serum/Plasma Advanced Kit (Qiagen) followed by reverse transcription using TaqMan^TM^ Advanced miRNA cDNA synthesis kit (Thermo Fisher Scientific) according to manufacturer’s instructions. For each serum sample, synthetic Caenorhabditis elegans miR-39 was added as a spike-in normalizer. To quantify the synthesized cDNAs, TaqMan MicroRNA assays were performed using ViiA7 Real-Time PCR System (Thermo Fisher Scientific).

### Statistical analysis

To analyse the RT-qPCR validation results, we used ddCT method^20^ to normalize and calculate the relative expression of selected candidate miRNAs. Statistical significance between groups was then analyzed with unpaired t-test utilizing Graphpad Prism 7. ClustVis^21^ was used to perform the Hierarchical Clustering and Principal Component Analysis (PCA) with normalized read counts data from HTG EdgeSeq system.

## Results

To identify the circulating miRNA pool from Fabry patients, 6 FD patients and 31 FD patients were recruited as screening cohort and validation cohort, respectively. The clinical characteristics of all patients were summarized in Table 1. At the time of visit there is no significant difference between ERT treated and ERT-naïve patients in age (p = 0.23), IVsd (p = 0.32), MSSI score (p = 0.1) and the ratio of mutation types (p = 0.46 by Fisher’s exact test), while the lyso-Gb3 and eGFR in ERT treated group were significantly lower than ERT-naïve patients with p = 0.02 and p = 0.01 respectively. Among the ERT-naïve patients visited in our study, 2 out of 3 in screening cohort, and all 14 in validation cohort were treated with ERT afterwards.

**Figure 2:**
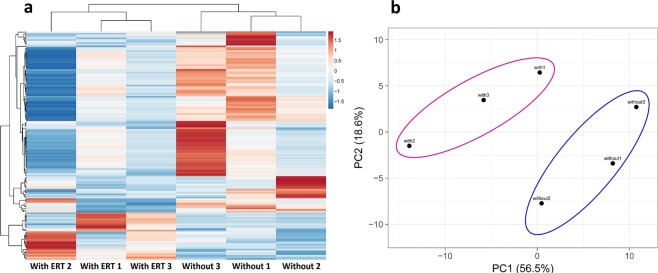
The overall expression pattern of regulated miRNAs. (**a**) Heatmap illustrates the differentially expressed miRNAs in serums of Fabry patients with and without ERT. Rows (expression level of miRNAs) and columns (serum samples) are clustered using correlation distance and average linkage. (**b**) PCA plot of the miRNA expression data indicates the distance between serum samples. X and Y axis show principal component 1 and principal component 2 that explain 56.5% and 18.6% of the total variance, respectively. Each dot in the plot represents one of the six samples used for sequencing based screening.

By using this innovative extraction-free HTG EdgeSeq system and intensive bioinformatical analyses, 296 miRNAs were detected in at least 4 out of 6 serum samples from Fabry patients (Fig. 1b); among them 269 miRNAs were expressed in both conditions; 145 miRNAs were found to be regulated more than 1.5 fold independent of p-value (Table 2). In addition, the overall expression pattern of the deregulated miRNAs decently distinguishes between the serums of Fabry patients with and without ERT by Hierarchical Clustering and Principal Component Analysis (Fig. 2).

**Table 2:** Top 100 Circulating miRNAs detected by RNA-seq based screening.

miRNA ID	Average expression level^a^	Fold change	p-value^b^
miR-197-5p	4344.69	25.07	0.22
miR-4739	7930.01	17.38	0.20
miR-1287-5p	1022.23	10.20	0.25
miR-4741	1580.09	9.02	0.21
miR-4633-3p	502.73	−5.57	0.16
miR-4516	10597.82	4.50	0.18
miR-7107-5p	319.39	4.26	0.01
miR-4316	38887.06	4.21	0.31
miR-3141	276.24	3.82	0.15
miR-1255b-2-3p	672.50	3.75	0.29
miR-4651	4219.61	3.69	0.26
miR-940	238.90	−3.62	0.06
miR-6084	185.83	−3.36	0.19
miR-3197	2062.72	−3.19	0.38
miR-4443	655.07	−3.15	0.45
miR-6729-5p	2790.31	−3.10	0.05
miR-19b-3p	331.55	−2.95	0.27
miR-4792	9380.74	−2.83	0.27
miR-663a	4894.42	2.82	0.04
miR-3178	4778.00	−2.81	0.27
miR-23a-3p	180.03	−2.79	0.27
miR-26a-5p	257.18	−2.42	0.30
miR-6124	447.85	2.39	0.02
miR-6891-5p	3078.59	2.35	0.33
miR-6089	8881.77	−2.34	0.45
miR-126-3p	307.41	−2.32	0.21
miR-6131	3109.86	−2.30	0.34
miR-339-3p	253.52	−2.29	0.27
miR-4638-3p	637.50	−2.26	0.29
miR-149-3p	481.19	2.24	0.28
miR-4479	209.75	−2.24	0.08
miR-6087	1160.67	2.23	0.05
miR-6510-5p	514.82	−2.21	0.13
miR-4497	8499.77	−2.20	0.24
miR-6512-3p	1196.18	−2.17	0.32
miR-548d-5p	266.18	2.16	0.38
miR-19a-3p	129.45	−2.12	0.27
miR-4469	309.30	−2.12	0.18
miR-541-3p	366.03	−2.11	0.05
miR-7158-5p	372.75	−2.10	0.32
miR-638	4809.08	−2.08	0.18
miR-21-5p	148.52	−2.07	0.19
miR-4433b-5p	957.59	−2.07	0.10
miR-6512-5p	4124.62	−2.05	0.32
miR-6727-5p	256.21	2.05	NA
miR-1973	267.03	−2.05	0.24
miR-1181	1220.70	−2.04	0.07
miR-548at-5p	822.04	−2.01	0.38
miR-1286	1237.36	−2.01	0.26
miR-4787-3p	1609.37	−2.00	0.05
miR-2277-5p	236.23	−2.00	0.25
miR-4634	394.67	−1.98	0.09
miR-3151-3p	331.77	−1.97	0.29
miR-1273c	162.68	1.96	0.23
miR-486-5p	331.51	−1.94	0.39
miR-1245a	561.57	−1.94	0.38
miR-223-3p	407.46	−1.93	0.36
miR-4285	431.48	−1.93	0.09
miR-6789-5p	170.97	−1.93	NA
miR-152-5p	512.10	−1.91	0.11
miR-6732-3p	198.95	−1.91	0.05
miR-4534	169.87	1.90	NA
miR-210-3p	125.42	−1.90	0.10
let-7a-5p	175.29	−1.90	0.27
miR-6798-3p	1147.64	−1.89	0.08
miR-548at-3p	641.72	−1.87	0.34
miR-6746-3p	297.65	−1.87	0.25
miR-582-3p	396.36	−1.86	0.35
miR-6876-5p	181.81	−1.84	NA
miR-7855-5p	1164.79	−1.83	0.10
miR-6796-3p	295.95	−1.83	0.08
miR-185-5p	181.51	−1.82	0.20
miR-8072	569.10	1.81	0.06
miR-6730-3p	394.81	−1.81	0.34
miR-764	1195.93	−1.80	0.09
miR-1273h-5p	3464.16	1.80	0.27
miR-92a-3p	412.94	−1.80	0.37
miR-148a-5p	3475.90	−1.79	0.32
miR-561-3p	327.03	−1.79	0.26
miR-1307-3p	2669.88	−1.79	0.11
miR-4461	118.37	−1.79	NA
miR-6085	156.10	−1.79	0.10
miR-4284	374.30	−1.78	0.27
miR-6836-3p	789.31	−1.78	0.07
miR-3960	63948.48	−1.78	0.48
miR-8073	11211.83	−1.77	0.12
miR-8075	1564.40	−1.77	0.12
miR-4784	346.98	−1.76	0.12
miR-6870-3p	1492.88	−1.76	0.17
miR-326	341.75	−1.76	0.12
miR-7847-3p	270.00	1.75	0.05
miR-6077	1161.12	−1.75	0.25
miR-1273a	149.86	1.75	0.19
miR-4271	487.17	1.75	0.30
miR-762	999.82	−1.74	0.53
miR-6790-3p	257.52	−1.74	0.14
miR-1307-5p	247.21	−1.74	0.04
miR-6727-3p	188.30	−1.74	0.53
miR-1251-5p	244.22	−1.74	0.10
miR-8064	8581.74	−1.73	0.17

^a^Average value of normalized miRNA read counts.

^b^*p*-values were calculated by unpaired two tailed t-test. NA: not available.

Of interest many miRNAs were detected by the high-throughput approach for which no clear role in biology or pathophysiology has been described yet. However, some miRNAs were already known in the literature. For instance, overexpression of miR-541 promote vascular smooth muscle proliferation and invasion suggesting that lower miR-541 levels might be beneficial in various vascular and pulmonary diseases^22^. Specific inhibition/silencing of miR-21 have been proved to be able to effectively prevent the myocardial and renal fibrosis^14,23^. The miR-17-92 family that comprises miR-17, miR-18a, miR-19a, miR-19b-1, miR-20a, and miR-92a-1 has been implicated in the promotion of cell proliferation and the growth of renal cysts^24^. Reduced levels of miR-26a were observed to be correlated with kidney injury in renal vascular disease and the restored expression could attenuate interstitial fibrosis and tubular apoptosis hence rescuing the renal function^25^.

**Figure 3:**
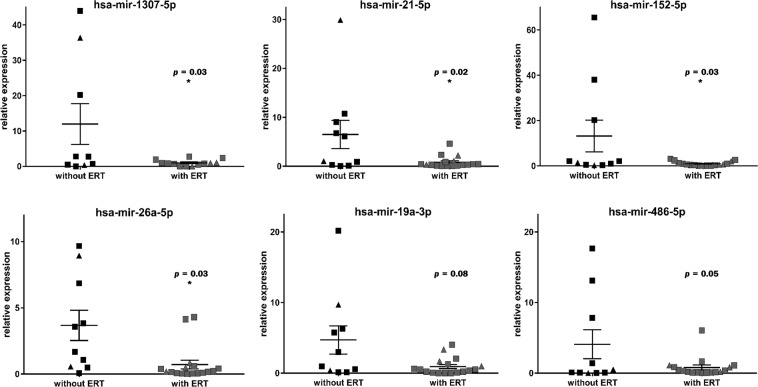
Validation of miRNA candidates in serum of Fabry patients. Illustration of the relative expression of miRNA candidates validated in serum samples of Fabry patients by RT-qPCR. Data from female patients and male patients are presented by triangles and squares, respectively. *p < 0.05.

Taken together with the differential expression evidence from our sequencing-based profiling results and the published data of characterized miRNAs, we selected 9 candidate miRNAs (miR-1307-5p, miR-541-3p, miR-4787-3p, miR-21-5p, miR-152-5p, miR-19a-3p, miR-19b-3p, miR-26a-5p, and miR-486-5p) from the top 100 deregulated miRNAs (Table 2) to perform RT-qPCR with serum samples in a validation cohort (n = 31; 17 with ERT and 14 without). As results, 4 miRNAs, miR-1307-5p, miR-21-5p, miR-152-5p and miR-26a-5p were found to be significantly (p < 0.05) down-regulated in the serum of Fabry patient after ERT (Fig. 3). MiR-19a-3p and miR-486-5p were also decreased but not significantly.

**Figure 4:**
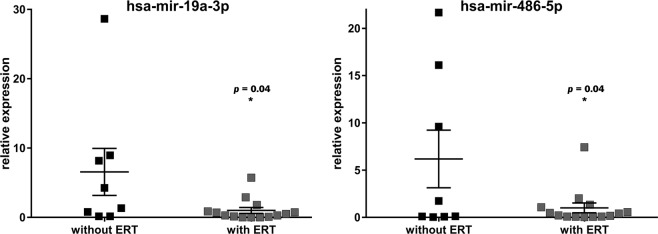
Validation of miRNA candidates in serum of male Fabry patients. Illustration of the relative expression of miRNA candidates validated in serum samples of male Fabry patients by RT-qPCR. *p < 0.05.

Since Fabry disease is an X-chromosome linked genetic disorder that affects male patients more severely than female, we made an additional analysis to compare the expression level of candidate miRNAs in 26 serums of male patients (14 with ERT and 12 without). Of interest two additional miRNAs, miR-19a-3p and miR-486-5p were found to be significantly (p < 0.05) down-regulated in male patients with ERT (Fig. 4). These findings are consistent with the facts that female Fabry patients demonstrate more variable symptoms with a wider range of disease severity^26^ and suggest that a gender specific miRNA-expression pattern is necessary to develop the optimal markers for female and male patients, respectively.

## Discussion

Although efficacy and clinical effects of ERT in patients with Fabry disease have been investigated and reported^5–8^, less is known about the mechanism and effect on the molecular level. In this study we performed a direct comparison of the miRNA expression pattern between patients with and without ERT that provide novel ideas to unravel the pathway underlying ERT.

**Figure 5:**
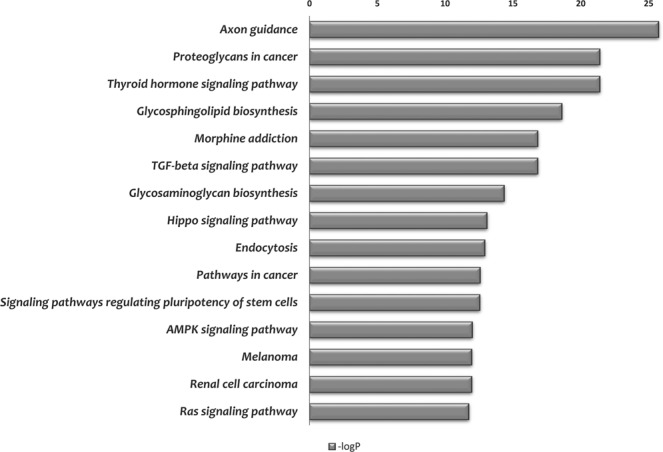
Pathway analysis of the deregulated miRNAs. Significant targeted KEGG pathways identified by top 100 deregulated miRNAs. X-axis indicates the log transformed p-value (significant level) between miRNAs and each pathway.

To elucidate the putative underlying molecular mechanisms, mirPath^27^ was utilized to make pathway enrichment analysis based on top 100 deregulated miRNAs. Of interest, axon guidance and TGF-beta signaling pathways were found to be targeted by the miRNAs (Fig. 5). Although improvement of small nerve fibre function with decreased neuropathic pain has been reported in FD patient with ERT^28^, the pathogenesis of the peripheral neuropathy correlated with Fabry disease is poorly understood. The predicted functional changes in axon guidance molecules caused by dysregulated miRNAs could affect the neural circuits developments that result in neurological symptoms in FD patients.

Renal impairment is often observed in later stage of Fabry disease, which advances to kidney failure causes significant mortality in FD patients. Improvement and slowing of the renal disease progression have been reported after ERT treatment^29^. More recently, proteomic studies demonstrated that VEGF receptor-2 in plasma of patients was significantly higher than controls and decreased after ERT^12^; increased expression TGF-β1 and VEGF were found to be associated with the renal pathogenesis of Fabry mouse model^30^. These findings suggest a putative function of TGF-β signaling pathway involved in nephropathy of Fabry disease, which is in general consist with our result from pathway enrichment analysis.

On the other hand, evaluation of the circulating miRNAs as biomarkers have been performed either in the field of kidney disease or Fabry disease. The concentration of circulating miRNAs in plasma including miR-21 and miR-210 were found to be reduced in patients with chronic renal failure, while no correlation was observed between urinary miRNAs and kidney function^31^. In a recent case study of a young Fabry patient without nephropathy manifestations, the expression level of miR-29 and miR-200 were found to be decreased in urinary sediment while the other TGF-β related miRNAs not^32^. Taken together, although TGF-β signalling pathway was suggested to be associated with Fabry nephropathy^12,28^, there is no direct evidence to support the putative involvement of TGF-β regulated miRNAs in ERT treatment.

In our study, a non-biased approach based on high-throughput sequencing were applied instead of knowledge based candidates selection. Although some known TGF-β related miRNAs e.g. miR-29, miR-192 and miR-200 were excluded from further validation due to the extremely low abundance in screening result, our result from pathway enrichment analysis still successfully predicted many miRNAs including miR-21-5p and miR-19a-3p that involved in the TGF-β signalling pathway. Although there were only 6 samples used in the screening step, we have proved the expression changes of miRNA candidates in additional 31 serums. The whole strategy applied in this study is based on a robust but unbiased approach from the technique to the data analysis.

However, the small size of studied population, selection bias (males and females with variable Fabry phenotypes), and the fact that circulating miRNAs from serum could come from various cell types and tissues are obvious limitations of this study. As the objects in this study are diagnosed Fabry patients, and our major aim is to identify miRNA pattern that involved in the beneficial effects of ERT, healthy control group were not included. Future studies including healthy controls could help to increase the specificity of our results to Fabry disease.

In conclusion, the resulting miRNA pattern together with the validated miRNAs are expected to improve the understanding of the mechanisms involved in the beneficial effects of ERT or potentially to identify Fabry patients with specific need for ERT. Further studies are needed in greater patient cohorts and proper controls.

## Data Availability

All data analysed during this study are included in this article. The datasets generated during the study are available on reasonable request.
